# The Role of Transoral Robotic Surgery in the Era of Hypoglossal Nerve Stimulation

**DOI:** 10.3390/jcm12134532

**Published:** 2023-07-06

**Authors:** Luigi Marco Stringa, Claudio Vicini, Giovanni Cammaroto

**Affiliations:** ENT Unit, Morgagni Pierantoni Hospital, 47121 Forlì, Italy; luigi.stringa@auslromagna.it (L.M.S.); claudio@claudiovicini.com (C.V.)

Obstructive sleep apnea (OSA) is a sleep-related breathing disorder characterized by repeated collapses of the upper airway walls, leading to a complete or partial reduction of airflow. This can result in oxygen desaturation and arousal during sleep.

Although OSA has been proven to be associated with serious health and social problems, it is still underestimated. Its prevalence in the general population is estimated to be approximately 24% among men and 9% among women.

Considering the impact of OSA on general health conditions, it has been proven that continuous desaturations are independent risk factors for hypertension, myocardial infarction, and stroke. OSA has been reported to be related to type II diabetes, metabolic syndrome, Parkinsonism, Alzheimer’s disease, and dementia [1].

Some other effects of OSA include insomnia and daytime sleepiness. The latter condition has been shown to be a significant social problem. It has been demonstrated that people with OSA have an increased risk of motor vehicle crashes compared to the general population, thus posing a problem for both themselves and others [2].

In order to establish a diagnosis, a thorough medical history, evaluation of the Epworth Sleepiness Scale, and objective assessment of breathing during sleep using polysomnography are essential. Additionally, other examinations are suggested for planning the most effective therapeutic strategy. Drug-induced sleep endoscopy (DISE) can be helpful in understanding which parts of the upper airway walls collapse and to what extent they are involved [1]. Similarly, lateral teleradiography can be performed to evaluate the presence of various cephalometric parameters. Certain anatomical variables, such as mandibular length, length of the uvula and soft palate, perpendicular distance between the mandibular plane and hyoid point (MPH), and distance between the posterior pharyngeal wall and posterior lingual surface (PAS), are known to be strongly correlated with OSA [3,4]. Surgical modifications of one or more of these parameters may represent an effective treatment for this condition.

There are various options available for treating OSA, each with different efficacy, invasiveness, and indications. The simplest measure is represented by weight loss, which not only reduces cardiovascular and metabolic risks associated with OSA but also improves pharyngeal obstruction by reducing the fat proportion in the upper-airway walls [5].

Other conservative approaches include positional therapy (encouraging side sleeping or elevating the head and trunk by 30°), the use of mandibular advancement devices (MAD), and continuous positive airway pressure therapy (CPAP), which remains the gold standard treatment [1,6]. Weight loss can be challenging to achieve and often takes time to show effectiveness. Positional therapy is only suitable for a subset of patients and can be difficult to achieve and uncomfortable, resulting in reduced adherence. CPAP therapy is highly effective but not well tolerated by many patients. It has been estimated that less than 50% of patients use CPAP for more than 4 h per night, nullifying its effects. In this regard, MADs offer similar effectiveness with better tolerance than CPAP, making them a good alternative to positive pressure therapy. However, MADs have limitations, as they may only be effective in mild and moderate cases of OSA and require the assistance of a qualified dentist for device fabrication [7].

Furthermore, surgical options can be considered, especially when other treatments are ineffective or poorly tolerated. Surgery offers a highly effective opportunity to treat OSA, improving the quality of life and avoiding dependence on mechanical devices for the rest of one’s life [8]. However, it is crucial to thoroughly evaluate the application of surgery, as OSA patients are vulnerable and typically have underlying health conditions that can increase the risk of complications. In this regard, factors such as male gender, diabetes mellitus, ASA score ≥ 4, and elevated alkaline phosphatase (ALP) values are predictive of medical and surgical complications following OSA surgery [9].

Different surgical techniques can be applied based on the cause and location of upper airway collapse. The base of the tongue (BOT) is one of the anatomical sites implicated in the obstructive pattern in patients with OSA, and trans-oral robotic surgery (TORS) is a well-documented and effective surgical option. The goal of this approach is to reduce the volume of the base of the tongue to restore a proper airway space with low surgical invasiveness in relation to the anatomical region. Depending on patient characteristics, particularly the histology and geometry of obstructive tissue, different surgical choices can be considered.

If the lymphoid tissue of the tongue is the primary obstructive component, removal of this hypertrophic tissue through “lingual tonsillectomy” is the most suitable option. More frequently, both lymphoid and muscular tissues contribute to an enlarged BOT, and in these cases, a “posterior middle glossectomy” or “tongue base reduction” is required to simultaneously remove lymphoid and muscular tissues. In addition to these approaches, a supraglottoplasty can be performed as an optional stage if floppy and/or redundant epiglottic, aryepiglottic, and/or arytenoid mucosa are responsible for airway collapse during inspiratory phases. If the sites of obstruction are at different levels of the upper airway, TORS can be combined with other surgical procedures, such as septoturbinoplasty, expansion sphincter pharyngoplasty (ESP), barbed relocation pharyngoplasty (BRP), and uvulopalatopharyngoplasty (UPPP).

There are some limitations in the application of TORS, such as a mouth opening measured as an interincisive distance less than 25 mm and a body mass index (BMI) > 30 kg/m^2^. These conditions have been shown to increase the rate of surgical failure. Although robotic surgery is a minimally invasive technique, it is associated with some complications. The most common post-operative complication is transient dysphagia, which does not seem to be related to the volume of tissue removed. Postoperative bleeding has been reported in 4.2% of cases. Transient hypogeusia, pharyngeal edema, and globus sensation have also been described as consequences of the compressive action of the tongue blade on lingual nerves and the pharyngeal wall or due to exposure to the high temperature of the monopolar cautery.

A more recent surgical option for the treatment of OSA is upper airway stimulation (UAS). This approach involves the use of electrical stimulation of the distal portion of the hypoglossal nerve (HN), resulting in muscular contractions, particularly of the genioglossus and geniohyoid muscles. The contraction of these muscles during sleep induces tongue protrusion, ensuring upper airway patency. Currently, two techniques are recognized. The first technique involves monolateral stimulation of the HN by using a subcutaneous generator installed in the chest. Two additional incisions are required for the placement of electrodes for HN stimulation in the submandibular region and for a pleural respiratory sensor in the chest. A more recent device allows bilateral stimulation of the HN through a single submental incision by using an external activation unit placed under the chin. This technique allows for a less invasive installation and eliminates the need for a generator inside the body. Currently, UAS is indicated for patients with moderate to severe OSA who cannot tolerate or have failed positive airway pressure and are over 22 years of age. Patients with a body mass index (BMI) greater than 32 kg/m^2^ and a complete concentric collapse pattern of the palate are associated with lower therapeutic success using UAS. Complications associated with HN stimulators include infections, device expulsion through the skin, pleural and neural injuries, malfunctions related to electronic components, tongue abrasion, and mouth dryness. Despite these drawbacks, UAS has been proven to be effective in treating moderate to severe OSA patients and reducing self-reported daytime sleepiness [10]. The therapeutic effects of UAS can be compared to a more invasive procedure such as maxillomandibular advancement, although with reduced efficacy. This makes UAS a valid solution for patients with high surgical risks [11].

TORS and UAS are two effective approaches for the treatment of OSA, and to date, their results have not been compared in the literature. Although more research is needed to clarify the role of these techniques, UAS shows promising results and low invasiveness, which will likely increase its application for sleep apnea treatment, especially in complex cases. However, the application of TORS will be crucial for OSA patients characterized by hypertrophic BOT, in which more conservative approaches are ineffective.
